# PrionScan: an online database of predicted prion domains in complete proteomes

**DOI:** 10.1186/1471-2164-15-102

**Published:** 2014-02-05

**Authors:** Vladimir Espinosa Angarica, Alfonso Angulo, Arturo Giner, Guillermo Losilla, Salvador Ventura, Javier Sancho

**Affiliations:** 1 Departamento de Bioquímica y Biología Molecular y Celular, Facultad de Ciencias, Universidad de Zaragoza, Pedro Cerbuna 12, 50009 Zaragoza, Spain; 2 Institute for Biocomputation and Physics of Complex Systems (BIFI), Universidad de Zaragoza, Mariano Esquillor, Edificio I + D, 50018 Zaragoza, Spain; 3 Joint Unit BIFI-IQFR (CSIC), Serrano 119, 28006 Madrid, Spain; 4 Institut de Biotecnologia i de Biomedicina, Universitat Autònoma de Barcelona, 08193 Bellaterra, Spain; 5 Departament de Bioquimica i Biologia Molecular, Universitat Autònoma de Barcelona, 08193 Bellaterra, Spain

**Keywords:** Prion domain, Protein aggregation, Amyloid fibrils, Prion prediction

## Abstract

**Background:**

Prions are a particular type of amyloids related to a large variety of important processes in cells, but also responsible for serious diseases in mammals and humans. The number of experimentally characterized prions is still low and corresponds to a handful of examples in microorganisms and mammals. Prion aggregation is mediated by specific protein domains with a remarkable compositional bias towards glutamine/asparagine and against charged residues and prolines. These compositional features have been used to predict new prion proteins in the genomes of different organisms. Despite these efforts, there are only a few available data sources containing prion predictions at a genomic scale.

**Description:**

Here we present PrionScan, a new database of predicted prion-like domains in complete proteomes. We have previously developed a predictive methodology to identify and score prionogenic stretches in protein sequences. In the present work, we exploit this approach to scan all the protein sequences in public databases and compile a repository containing relevant information of proteins bearing prion-like domains. The database is updated regularly alongside UniprotKB and in its present version contains approximately 28000 predictions in proteins from different functional categories in more than 3200 organisms from all the taxonomic subdivisions. PrionScan can be used in two different ways: database query and analysis of protein sequences submitted by the users. In the first mode, simple queries allow to retrieve a detailed description of the properties of a defined protein. Queries can also be combined to generate more complex and specific searching patterns. In the second mode, users can submit and analyze their own sequences.

**Conclusions:**

It is expected that this database would provide relevant insights on prion functions and regulation from a genome-wide perspective, allowing researches performing cross-species prion biology studies. Our database might also be useful for guiding experimentalists in the identification of new candidates for further experimental characterization.

## Background

Prions are a special type of amyloids, which can act as heritable elements in their aggregated state, constituting self-replicating entities that can perpetuate and transmit over generations. Prions are generally ubiquitous proteins with specific functions when folded but, after their amyloid conversion, they also perform important functions in cells, acting as epigenetic elements [1,2], evolutionary capacitors [3,4] and bet-hedging devices [5,6] in the processes of adaptation to environmental fluctuations in microorganisms, and in mechanisms crucial to maintain long-term physiological states in invertebrates [7-9]. Despite these beneficial roles in cell physiology, prion formation is more commonly thought to be associated with disease, due to the growing number of serious and in some cases incurable pathologies caused by the deposition of prion fibrils, comprising a diverse group of neurodegenerative disorders in humans and mammals [10-16]. Notwithstanding their important role in cell physiology and pathology, the number of prions known so far is scarce, and little is known regarding their implication in the regulation of cellular processes from a genomic perspective. The main motivation for the construction of PrionScan is to disclose and make available to the scientific community the most extensive set of putative prion-forming proteins, predicted for all the proteins encoded in the genomes of all the organisms annotated in public databases.

The particular structural and primary sequence characteristics of prion domains have been used to try to predict the prionogenicity of proteins. Among amyloids, prions stand out for their high content of the polar residues glutamine and asparagine, which lowers the success rate of the traditional algorithms designed to identify aggregation-prone amyloidogenic regions in protein sequences [17-20], since prion domains do not share the sequential characteristics common to *β*-sheet-amyloid forming regions [21]. Although the strong compositional bias of prion domains towards glutamine and asparagine has been used to make predictions at a genomic scale [22,23], it has not been until recently that the increase in the number of known prion sequences has allowed the construction of more accurate predictive models. Based on these compositional characteristics, prion-like sequences have been underscored at a genome scale in yeast [24]. Other studies relying on the aggregation of variants of the yeast prion *Sup35p* when expressed *in vivo* have rendered compositional models successfully used to score protein sequences on the basis of their prionogenicity [25,26]. From the most extensive set of experimentally tested prion and non prion sequences in yeast [24] we have generated a probabilistic model of Q/N-rich prionogenic regions that has been thoroughly benchmarked to handle large sequence databases, yielding a fairly good predictive performance [27], and used it to predict prion-like proteins in all the complete proteomes available in public databases.

Our methodology is based on the amino acid propensities extracted from a set of 29 yeast protein sequences for which there is strong experimental evidence of prion formation *in vivo* and *in vitro*, and a set of 18 sequences included in the same study that share similar compositional characteristics with the other 29 prions, but showed no prion behavior in the same experimental tests under similar conditions [24]. Those sequences were used to build the probabilistic model and benchmark it to assess the performance at rescuing real prions from non-prions, with an area under the ROC plot for the test of 0.90. We defined the length of a prion domain to be 60 contiguous residues, and set up a sliding-window algorithm to scan protein sequences from end-to-end. We also set up an assay to evaluate the performance of our model to handle large datasets of protein sequences, by scanning three negative test sets of protein sequences yielding recovery values of almost 90% of the true positives with precision values above 80%, and an evident independence of the results from the number of negative instances in the scanned datasets [27]. These fairly good predictive results somehow validate the predictions we obtained in the complete proteomes of organisms, which uncovered a large set of proteins bearing domains with high compositional similarities to *bona fide* prions. The preliminary analysis performed using the large amount of new data generated in this study, revealed some interesting trends in the distribution of putative prion proteins in functional families, related to different biological processes and localized in specific cellular components depending on the taxonomic subdivision and the specific organism analyzed [27].

Given the need for predictive tools that can forecast protein prionogenicity at a genomic scale to guide experimentalists, and also to provide a global view of the relevance of prions for the regulation of cellular processes, we decided to build PrionScan, as an open source of up-to-date prion predictions for all the proteins annotated in public databases. The complete system is updated on a four-weekly basis following the update of UniprotKB [28], to include the predictions for the most recent releases of sequences, either curated entries from Swissprot or sequences automatically generated from massive sequencing programs in TrEMBL. The present version of PrionScan includes detailed information for 27925 putative prion proteins in 3236 organisms from almost all taxonomic subdivisions. Aiming at providing the scientific community with a highly functional site for the study of prion biology, we designed a simple and flexible querying system suitable for data mining by combining different sorts of information included in our database to recover, for instance, prion predictions in the complete genome of an organism or for proteins belonging to a specific functional family or related to a specific biological process. To complete the functionality of our service, we also set up a bundle to our statistical model that provides an easy way of analyzing a large number of protein sequences not reported in public databases, for example mutants of existing proteins, *de novo* synthetic species or yet-to-annotate sequences.

## Construction and content

### Data acquisition and database organization

Our main source of information is UniprotKB [28], the standard and most complete repository of protein sequences freely available. Following each update of this database once a month, we thoroughly scan all the entries included both in Swissprot and TrEMBL in the search for prion-like domains according to our methodology, as previously described elsewhere [27]. In parallel, we also extract some relevant information from UniprotKB for those entries containing putative prion domains, and store it in our database. The data generated during the prediction process comprises the score of the highest scoring window during the scan of a protein sequence, the sequence of the highest scoring domain, the localization of the highest scoring putative prion-domain and the complete scanning profile of the protein sequence, which are merged with the information extracted from UniprotKB entries, including the entry identifier and accession number, the organism and taxon names, the protein names, the Gene Ontology [29] GO Terms for the molecular functions, biological processes and cellular components in which the protein is related/located and finally, cross-references to other databases with relevant information for the protein bearing putative prion domains.

### Database content

**Figure 1:**
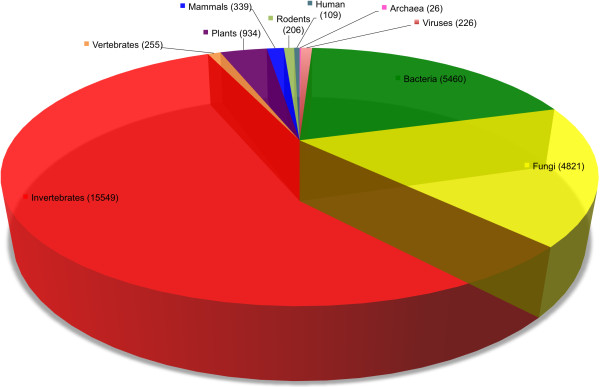
**Distribution in all taxa of the predictions compiled in PrionScan.** The pie chart depicts the distribution of prion predictions in all different taxa, from archaea to humans. The number of predictions in each taxon is shown in parenthesis. The predictions in each taxon are organized following the structure of UniprotKB [28] taxonomic subdivisions, in which the proteins in the taxa rodents, mammals and human are distributed separately.

All this information is stored in a MySQL database environment, allowing linking of all the information at all possible levels to enable the efficient querying of the database for fine-grained data retrieval. A description of the data in the present version of PrionScan, including the predictions for the UniprotKB (update 2013_09) of September 2013, is shown in Figure 1. This pie chart depicts the distribution of the 27925 proteins with prion domain predictions in the 3236 different organisms in all taxa from archaea to human. A comparison of these numbers with the predictions previously obtained in the initial paper describing our method, in which we used the UniprotKB (update 2012_02) from February 2012 [27], illustrates the increase in the number of predictions between these two versions in just twenty months, totaling an increment of more than 61% from the approximately 17400 predictions obtained at that time.

## Utility and discussion

### PrionScan website

**Figure 2:**
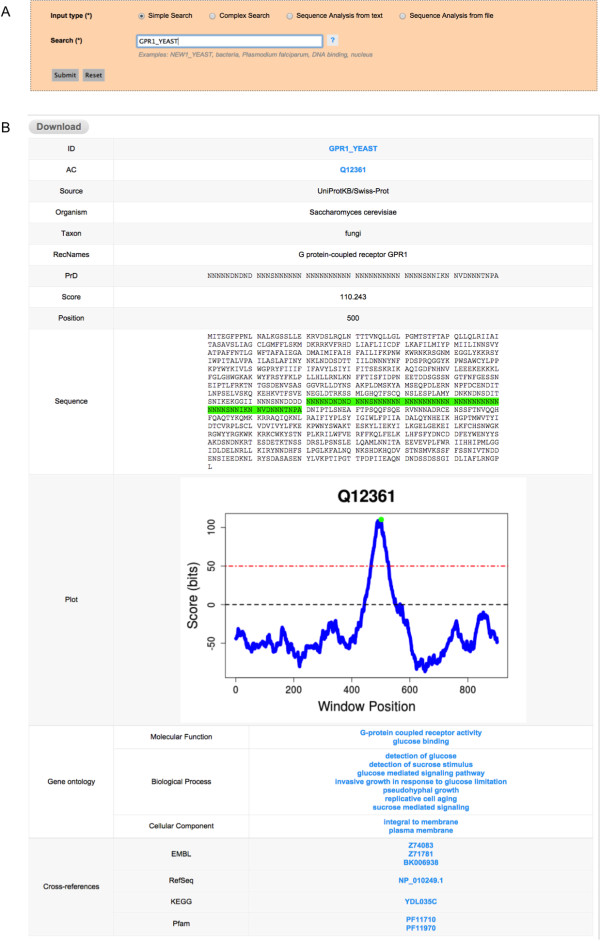
**The Simple Search option for direct access to protein prion prediction information. A)** The Simple Search option is used for querying the database using the UniprotKB identifier of a given protein. **B)** The Detailed Output Page retrieved by the query for a protein with putative prion domains. At the top there is a button for complete download of the results.

PrionScan is hosted in an Apache web server that relies on a PHP bundle to connect the client query patterns with the database and a set of *ad hoc* Perl scripts that perform some functions such as the prediction of prion domains in the client’s own sequences and the connection to our computer cluster for processing a large number of client sequences. The system processes the client searches and data submission and generates dynamic HTML pages designed to be completely functional in the most used web browsers. The home page of PrionScan contains a short introduction to our method and the functionalities of the site to guide the users in a glimpse, and the **Submission Form** organized in checkboxes to easily select the different searching alternatives (**Simple** or **Complex** Searches) and the two different ways of submitting sequences to be analyzed with our method (**Sequence Analysis** from **text** or **file**), please see Figure 2, panel A. There is also a link in the leftmost vertical menu to a page containing detailed help and guidance on the methodology, the searchable fields of the database and the output generated. Furthermore, in order to facilitate the use of our site without the requirement of a full reading of the Help page, we also enabled the auto completion utility in the **Simple Search** tab and added hover help buttons for in-site help.

### Querying the database

**Figure 3:**
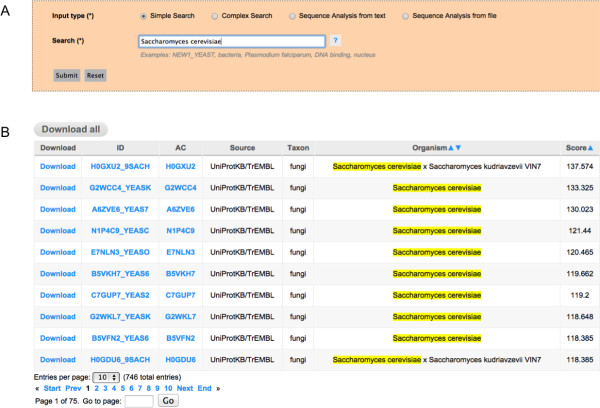
**The Simple Search option for searching with text keywords. A)** The Simple Search option is used for querying the database with a text keyword –i.e. the complete name of an organism in this case. **B)** The General Output Page retrieved by the query with rows corresponding to multiple entries in the database –i.e. putative prion proteins in the genome of this organism– each one redirecting to a Detailed Output Page. At the top there is a button for complete download of the results and at the bottom there is a summary of the number of predictions and a functionality for browsing throughout multiple pages containing all the results returned.

PrionScan is configured to be searched in two different ways:

•**Simple Searches:** The easiest way for retrieving information when the user wants to find out whether a specific protein contains prion-like domains. In this case it is possible to directly access the information of a single protein providing its UniprotKB identifier or principal **accession number**, as depicted in Figure 2, panel A. This option is also the best alternative for querying the database with information from one of the searchable fields **Taxon**, **Organism Name**, **Protein Name** (Recommended Name, Alternative Name and Submitted Name) and the Gene Ontology Terms for **Molecular Function**, **Biological Process** and **Cellular Component**. For example, it is possible to retrieve all the putative prion proteins in the genome of an organism by providing the complete or partial organism name, please see Figure 3, panel A.

**Figure 4:**
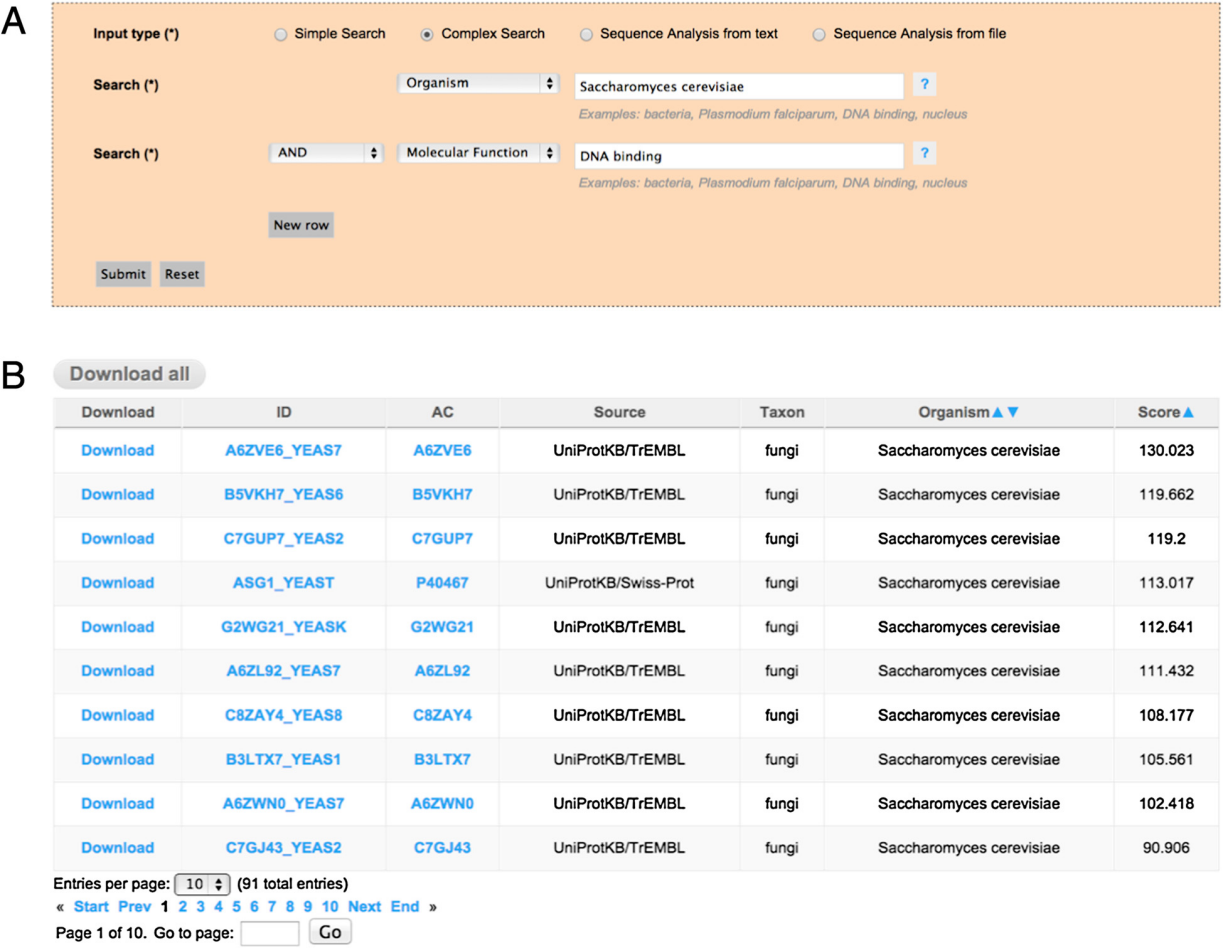
**Complex Search option for searching with multiple text keywords. A)** The Complex Search option is used for querying the database combining the information of two columns of the database –e.g. in this case we search the putative prions in the genome of an organism related to a specific molecular function as described in Gene Ontology. **B)** The General Output Page retrieved by the query with rows corresponding to multiple entries in the database –i.e. putative prion proteins in the genome of this organism– each one redirecting to a Detailed Output Page. At the top there is a button for complete download of the results and at the bottom there is a summary of the number of predictions and a functionality for browsing throughout multiple pages containing all the results returned.

•**Complex Searches:** Sometimes, however, more complex searches are needed, especially when the user has more detailed information of the set of proteins to be retrieved. In those cases the search can be refined by combining multiple fields from the database –*i.e.***Taxon**, **Organism Name**, **Protein Name** (Recommended Name, Alternative Name and Submitted Name) and the Gene Ontology Terms for **Molecular Function**, **Biological Process** and **Cellular Component**. These fields can be combined when needed, by introducing the search terms in the rightmost tabs, and selecting the appropriate field that should be considered in the leftmost tabs. You can also choose the logical operators combining the query instances. Using this option, it is possible, for example, to retrieve all the prion-like proteins having a similar Molecular Function or related to a specific Biological Process in the genome of a specific organism, as depicted in Figure 4, panel A.

•**The Output:** After performing a search for a specific protein using its UniprotKB **identifier** or principal **accession number**, if the protein selected has prion-like domains the output will be a **Detailed Output Page** including the UniprotKB identifier (ID) and principal accession number (AC), the source (Source) of the protein (coming from Swissprot or TrEMBL), the organism name (Organism) and taxon (**Taxon**), the names of the protein (recommended names: **RecName** and/or alternative names: **AltName** and/or submission names: **Subname**), the highest scoring prion domain in the sequence (**PrD**), the score of the highest scoring prion domain (**Score**), the position in the protein sequence of the highest scoring prion domain (**Position**), a representation of the complete protein sequence with the highest scoring prion domain highlighted in green (**Sequence**), and a graphical representation of the scanning of the complete protein sequence (**Plot**), corresponding to a chart with the score profile along the sequence, also showing the score used for making the predictions (Figure 2, panel B). In addition to these fields, the **Detailed Output Page** might also include information regarding the Gene Ontology Terms associated to the protein for the **Molecular Function**, **Biological Processes** and/or **Cellular Component** and the Cross-references to other databases like the EMBL, Refseq, Pfam and so on, lower part of Figure 2, panel B. However, if the search, either a **Simple Search** or a **Complex Search**, retrieves more than one entry, the output will be a **General Output Page** with columns and rows that could contain different information depending on the search conducted, with some columns enabled to be dynamically ordered in ascending or decreasing manner (Figures 3 and 4, panel B). Every row shown in this **General Output Page** redirects to a **Detailed Output Page** as described above. At the bottom part of the **General Output Page** we include a short summary of the number of results retrieved by the query, which is also useful for browsing forward and backwards to different pages in the **General Output Page** by using the page links, or just introducing the exact page in the ‘Go to page’ box (lower part of Figures 3 and 4, panel B). Independently of the type of query, it is possible to download the results retrieved in the form of a compressed file containing all the information displayed in the web version, which includes all the information of entries and the associate scanning plots. This information is in HTML format and can be displayed locally using any web browser, and we also include a version in a flat text file with the same information that could also be easily parsed by *ad hoc* scripts written by the users for performing in-house massive offline analysis of our data.

### Analyzing your own sequences

**Figure 5:**
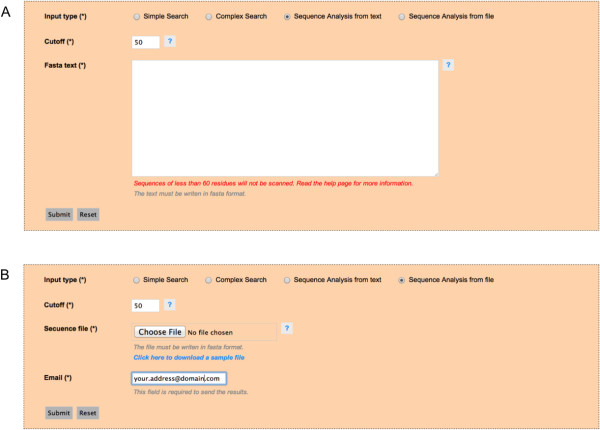
**Sequence Analysis from file and text for processing user’s own sequences. A)** The Sequence Analysis from text option in which the user can modify the cutoff used in our methodology to scan the sequences that can be pasted in the text box below. **B)** The Sequence Analysis from file option useful for processing a high number of sequences by submitting the file to be processed in our server. In this case there is an obligatory text box for providing the e-mail address for sending the results upon completion and the cutoff could also be adjusted at user’s discretion.

In this case the user has complete flexibility for testing the prionogenicity of protein sequences using the (**Sequence Analysis** from **text** or **file**) functionalities, as depicted in Figure 5. First, the right option in the **Submission Form** is selected in order to enable the option for pasting a limited number of sequences in FASTA format or for uploading a file with a high number of protein sequences, which can be either a flat file or a compressed file in FASTA format (the limit is 500 MB for compressed files, which we estimate can contain approximately one million sequences). We also provide the possibility that the user can select the best cutoff for prediction according to his/her needs. In this case, if only one among the sequences introduced by the user happens to bear prion-like domains, the output will correspond to a **Detailed Output Page** with the specific information for the protein. On the other hand if the analysis of the sequences results in more than one protein with prion domain predictions, then the output will be a **General Output Page** with one row for each protein with predictions. As in the case of results obtained while searching the database, each row redirects to a **Detailed Output Page** with the specific information for the selected sequence. If the number of sequences is less than 5000, the output will be generated in a few seconds in HTML format as just described here, but when the number of sequences is higher than this value, then the job will be submitted to our computer cluster for processing. In this last case the results will be submitted by e-mail to the user upon completion.

### Similar resources

There are a few examples of repositories with information on prion proteins, prionogenic sequences, prion-related diseases, prion protein interactions and orthologs and paralogs of prion proteins in multiple organisms. For example, the Prion Disease Database [30] contains a sort of experimental data on prion sequences and multi-level data on diseases caused by prions, combined with a set of tools for data analysis and systems biology studies in mouse. PrionHome [31] is a non-redundant database containing approximately 2000 prion-related sequences obtained from different public and private sources, in some cases with experimental support or inferred using different predictive algorithms [24,32,33]. There is yet another similar resource, set up as a web application for predicting prion forming propensity [25]. Though not a database in the strict sense of the term, the PAPA site (http://combi.cs.colostate.edu/supplements/papa/) allows the analysis of protein sequences based on amino acid propensities in prion sequences inferred from *in vivo* aggregation analysis. In contrast to these available resources, PrionScan provides genomic-scale prion predictions for the proteomes of all organisms, in a framework that allows an easy way to study the sequential/structural determinants of prionogenicity, as well as comparative studies of the implication of prions in cell biology in different group of organisms.

## Conclusions

The continuous growth in the number of protein sequences annotated in public databases, mainly due to massive genome sequencing programs, is challenging because the availability of experimental and computational methodologies for the analysis of those new sequences evolves at a rather slower pace. PrionScan intends to be a repository of organized and up-to-date predictive data on prion-like domains present in the proteins of all the organisms available. In this regard we believe that our database will provide a basis for future studies on the implication of prions in cell biology from a genomic perspective.

## Availability and requirements

PrionScan is publicly available in the following web address: http://webapps.bifi.es/prionscan

## Abbreviations

UniprotKB: Universal protein resource; TrEMBL: Transcription EMBL; GO: Gene ontology; PHP: Hypertext preprocessor; HTML: Hyper text markup language; SQL: Structured query language.

## Competing interests

The authors declare that they have no competing interests.

## Authors’ contributions

JS conceived the idea. VEA designed and implemented the database, wrote most of the code and performed all data processing. VEA and AA designed the web interface. AA set up the server and wrote the code for query processing, database functionality and update, and helped in data processing. GL, AG, SV and JS directed and oversaw the project. VEA, AA, SV and JS tested the user interface. VEA drafted the manuscript. AA, SV and JS helped correcting the manuscript. All authors have read and approved the manuscript.
